# Umbilical Incarcerated Hernia With Omental Eventration Caused by a Leech Bite in a Pediatric Patient

**DOI:** 10.7759/cureus.44831

**Published:** 2023-09-07

**Authors:** Carmel Sher, Yael Dreznik, Gal Yekutiel, Ron Berant, Dragan Kravarusic

**Affiliations:** 1 Department of Pediatric and Adolescent Surgery, Schneider Children’s Medical Center of Israel, Petah Tiqwa, ISR; 2 Faculty of Medicine, Tel Aviv University, Tel Aviv, ISR; 3 Department of Emergency Medicine, Schneider Children’s Medical Center of Israel, Petah Tiqwa, ISR

**Keywords:** omentum eventration, peritunium, alternative medical therapies, omentum evisceration, pediatric hernia, medicinal leech therapy

## Abstract

Medicinal leech therapy (MLT) is used in various medical disciplines, among which are reconstructive surgery and microsurgery. Medicinal leech therapy is also often adopted by alternative and traditional medicine, aiming to treat various common medical symptoms, such as fever and arthritis.

Congenital umbilical hernia is a rather common physical finding in the pediatric population, where every third Caucasian newborn, roughly, is being diagnosed with the condition, and even more so among the African population. Fortunately, most cases resolve spontaneously in the first years of life. Toddlers whose hernia does not close typically require umbilical hernia repair.

This article describes the case of a five-year-old girl with an asymptomatic congenital umbilical hernia who was admitted to the ER due to an omental eventration that occurred following the placement of a leech on her umbilicus in her parents’ attempt to treat a febrile episode. She subsequently underwent an urgent umbilical exploration and a repair of her umbilical hernia.

The main known risks of leeching are bacterial infection, anemia, prolonged bleeding, and, less frequently, pruritus, allergies, marked edema, and cellulitis. This article presents yet another possible complication that, to the best of our knowledge, has not been documented before in the literature.

Several old-school therapies transcended over time into medical disciplines. Given that "traditional" practices often take place within households and communities, it is of crucial importance to point out potential complications, both rare and common, that can be caused by those practices in order to reduce the risk of severe, undesired outcomes. Indeed, the growing interface between traditional, alternative therapies and modern, conventional medicine urges better parental guidance and improved education regarding potentially harmful and unauthorized interventions.

## Introduction

Medicinal leech therapy (MLT) or hirudotherapy is used in various medical disciplines, among which are reconstructive surgery and microsurgeries. It is often adopted by alternative and traditional medicine, aiming to treat various common medical symptoms, including, among others, fever and arthritis. As such, it is commonly classified as one of the old-school folk remedies applied in certain communities worldwide.

An ancient therapeutic technique, MLT exploits the saliva secreted by blood-sucking leeches during their feeding process. Leeches secrete more than 20 identified bioactive substances, such as hirudin, antistasin, and saratin, that have analgesic, anti-inflammatory, anticoagulant, and platelet inhibitory effects, as well as antimicrobial and thrombin regulatory functions [1]. In light of these broad-spectrum therapeutic capacities, MLT has been widely studied for possible positive effects, including wound healing and pain reduction, particularly in fields such as plastic, reconstructive, and microsurgeries [2-3].

In 2004, the United States Food and Drug Administration (FDA) approved the use of MLT for certain ailments. While officially limited to these fields only, the use of leeches for medicinal purposes, also known as "leeching", remains a popular therapeutic practice as a "home remedy" by traditional and alternative therapists [3]. This kind of therapy often includes placing the leech on different skin areas, for instance, on the umbilicus, aiming to lower body temperature upon a febrile attack. Yet, despite the longstanding use of MLT, our understanding of its safety is still insufficient, based primarily on retrospective studies and case reports. Indeed, current knowledge regarding possible complications of MLT is somewhat limited and includes infections, prolonged bleeding, and anemia, as well as other, less frequent complications. Publications of rare and unexpected complications are therefore scarce, despite their crucial importance. Hence this case report.

Congenital umbilical hernia is a very common physical finding in the pediatric population, affecting up to 30% of all White newborns and with an estimated prevalence as high as 85% among newborns of African descent. Fortunately, numbers decrease thereafter due to spontaneous gradual closure, with 2% to 20% at one year of age and less than 4% at four years of age [4-5]. The presence of an umbilical hernia in toddlers is typically a sufficient indication for umbilical hernia repair.

In this case study, we report a case of a child who presented with omental eventration following the placement of a leech on the child's umbilicus in an attempt by her parents to treat a febrile episode. The child was diagnosed with an umbilical incarcerated hernia and omental eventration and, subsequently, underwent an urgent umbilical exploration with a repair of the umbilical hernia. To the best of our knowledge, this is the first case of its kind to be documented in literature.

## Case presentation

**Figure 1:**
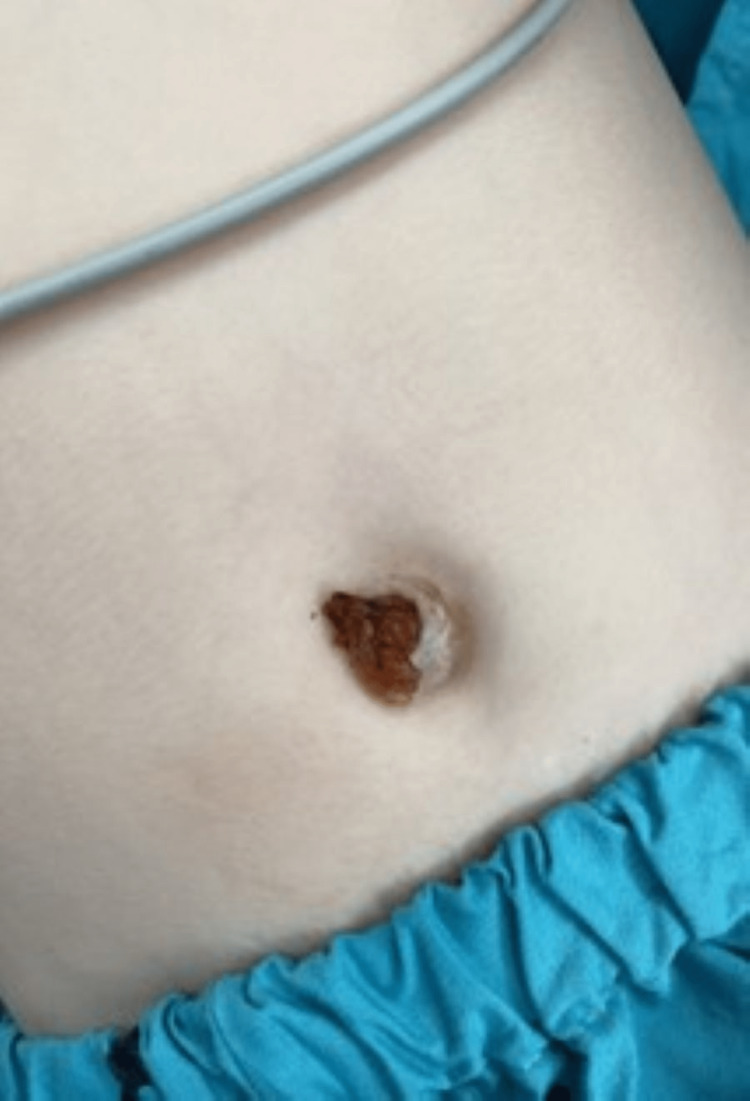
The patient’s abdomen has a visible protrusion of the omentum through the umbilicus.

A previously healthy five-year-old girl was admitted to our emergency department following a two-day fever that was attributed to otitis media by her general practitioner. Her parents chose to treat her fever with homeopathic remedies, along with the placement of a leech on her umbilicus. Indeed, they later revealed that such treatment is used frequently in their family. However, when they removed the leech, they were surprised to find visible tissue protruding out of her umbilicus (Figure 1), which led them to rush to our institute’s emergency room (ER).

The patient’s vital signs upon admission were as follows: blood pressure of 94/49 mmHg, heart rate of 138 bpm, temperature of 38.2 degrees Celsius, and 100% oxygen saturation in room air. Her physical examination revealed mild erythema of the left ear and an omental eventration in the center of the umbilicus, with signs of neither bleeding nor infection (Figure 1).

Blood work demonstrated a leukocytosis of 22,000 mm3 with 91% neutrophils and a C-reactive protein (CRP) of 16 mg/L. The patient was diagnosed with incarcerated umbilical hernia and omental eventration and was taken to an urgent umbilical exploration following the administration of broad-spectrum antibiotics, in accordance with our infectiologist’s recommendations.

**Figure 2:**
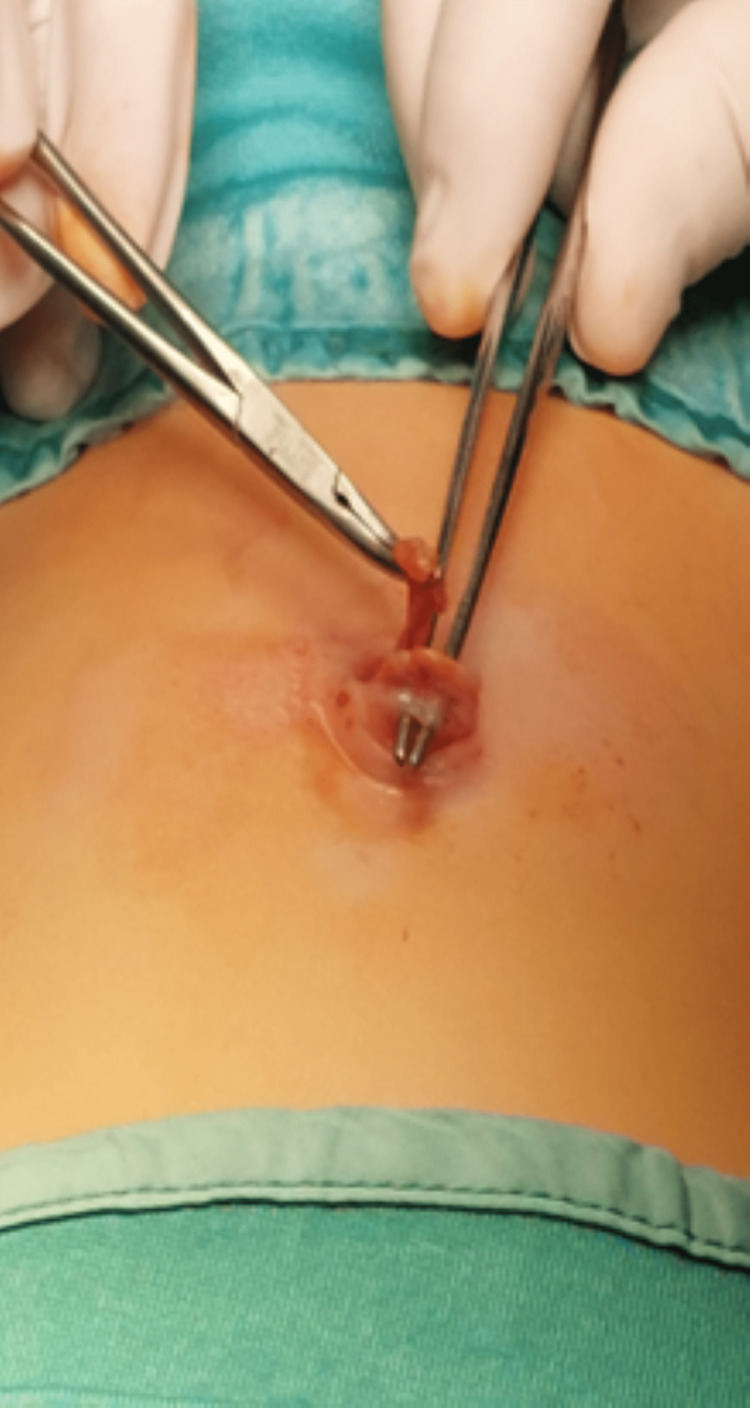
Intraoperative presentation of the umbilical hernia. Forceps are located in the bite-hall of the umbilicus, while mosquito forceps grab the omentum.

The surgery began with regular umbilical exploration (as common in umbilical hernia repair), following an incision below the umbilicus that revealed an umbilical hernia of roughly 4 mm defect, containing omentum that protruded through a 3 mm bite-hall in the umbilicus itself (Figure 2).

The omentum was tied, ligated, and then sent for bacteriology (with negative results reported). The umbilical hernia repair took place using absorbable sutures, after which the defect was closed. As for the umbilicus, it was closed by central stitches using absorbable sutures. The patient was discharged the following day with no other complaints or postoperative complications.

## Discussion

Current knowledge of common complications caused by leeching includes infections, anemia, and blood loss [6], with infectious incidence ranging from 4% to 20% [7]. Yet many other complications, albeit less frequent, were reported and documented throughout the years. These include pruritus, erythema, marked edema, nasal congestion, cellulitis, type IV hypersensitivity reactions, and others [3, 8]. Indeed, medical guidelines for the safe use of medical leeching are well described [9-10]. Having said that, both parents and alternative medicine providers are not always aware of the existence of such guidelines, nor do they attain sufficient knowledge regarding the potential risks of those procedures involving leeches. On top of that, most of these so-called "traditional" or "folk" therapies seldom interact with modern medicine, as they are typically applied within households and communities and often by unauthorized staff. Hence the importance of pointing out potential complications that can be caused by those practices.

This case is a unique description of a new complication, not previously documented, of leech therapy: an eventration of the omentum through a previously asymptomatic umbilical hernia, occurring as a result of a leech-bite, following an attempt to resolve a febrile episode in a five-year-old girl by placing the leech on her umbilicus.

Such a case of communication between the abdominal cavity and the outer atmosphere imposes a significant risk for serious complications, such as ileus, infections, sepsis, and, in severe cases, even death. Clearly, formal medical indications for leech therapy do not include the use of leeches as antipyretic agents, yet some people and certain communities do practice it, perhaps even on a regular basis. Such "therapy" can result, as described above, in an unwanted deep penetration of the leech that might, in turn, cause an eventration of the umbilical hernia sac along with its contents.

## Conclusions

Several old-school therapies transcended over time into medical disciplines. One of them, MLT, is being used in various medical procedures. The case reported hereinabove relates to the interface between such old-school therapies (leeching, in that case) and modern, conventional medicine. Given that "traditional" practices often take place within households and communities, it is of crucial importance to point out potential complications that can be caused by those practices in order to reduce the risk of severe, undesired outcomes. Leeching complications, such as the one described, underline the substantial importance of thorough parental guidance and professional communal education regarding potentially harmful and unauthorized interventions, such as the ones exploiting leeches, and in particular, those that include placing them on the umbilicus.
